# “Woulda, Coulda, Shoulda”. Workers’ Proactivity in the Association between Emotional Demands and Mental Health

**DOI:** 10.3390/ijerph16183309

**Published:** 2019-09-09

**Authors:** Greta Mazzetti, Silvia Simbula, Chiara Panari, Dina Guglielmi, Alessio Paolucci

**Affiliations:** 1 Department of Education Studies, University of Bologna, 40126 Bologna, Italy; 2 Department of Psychology, University of Milano-Bicocca, 20126 Milan, Italy; 3 Department of Economics, University of Parma, 43125 Parma, Italy

**Keywords:** mental health, emotional dissonance, proactivity, customer relations, emotions, retail distribution sector

## Abstract

The present study aimed to explore the mediating role of hostile customer relations in the association between emotional dissonance and workers’ mental health. Moreover, the moderating role of proactive personality as a buffer against hostile customer relations was assessed. Emotional demands become crucial within professions that involve a direct relationship with clients and, if poorly managed, can negatively affect workers’ health and performance. Accordingly, data were collected on a sample of *n* = 918 mass-retail employees working for one of the leading Italian supermarket companies. Most participants were women (62.7%) with a mean age = 40.38 (SD = 7.68). The results of a moderated mediation analysis revealed that emotional dissonance was related to more hostile customer relations that, in turn, were associated with higher rates of mental health symptoms. Proactive personality emerged as a protecting factor that prevented the onset of conflicts with clients, particularly among workers experiencing high levels of emotional dissonance. The identification of resources enabling management of emotional demands could suggest suitable adaptive strategies for customer-facing roles, thus preventing the occurrence of adverse mental health symptoms.

## 1. Introduction

In the last few decades, the European economic scenario has been characterised by a rapid expansion of the service sector, which became the prominent area of employment in the global economy [1]. Approximately 48% of the current workforce is employed in the service sector, and this rate is expected to grow as a result of labour market transformations and the prevalence of new technologies on a global scale [2]. Consistent with this international trend, in Italy the service industry accounts for more than 70% of the working population and more than 90% of people accessing the labour market works in this sector, which is the fastest growing of the national labour market [3]. The context of this study is represented by the retail trade sector, that currently accounts for almost 5% of the total value added of the European economies [4]. In particular, the Italian large-scale retail distribution nowadays counts almost 22 thousand commercial activities and almost half of them are supermarkets providing employment for over 400 thousand workers: in line with international trends, the sector reported an increase of 2.2% of sales areas all over the country [5].

A critical job demand in the service sector concerns the effective management of interpersonal relationships. Unlike other work sectors, service workers are required to deal with customers on a daily basis and, given the global economic competition of the labour market, a strategic objective for organisations requires improving the quality of customer experiences [6]. Constant face-to-face interaction with customers has increasingly emphasised the emotional dynamic involved, with academic and organisational literature paying close attention to emotional labour strategies applied by workers. Indeed, relationships with customers can represent a demanding aspect among service workers, who need to effectively manage their own emotions and feelings to ensure a positive customer experience [7]. To this purpose, workers must comply with the set of norms, labelled as emotional display rules, that organisations develop in order to define who is allowed to exhibit what specific emotional expressions, and under which circumstances [8]. Workers are socialised to these norms through formal policies, including the selection and training procedure, and through a system of informal social rewards and punishments. Emotional display rules could be observed in any professional role, but they become particularly relevant in the service sector where interpersonal interactions are involved. In this context, workers should exhibit specific emotions to customers as part of their job [9]. When workers perceive that the emotions they feel are incongruent with those they should display during customer interaction, they could adopt different emotional strategies in order to regulate their inner state and manifest behaviour [10]. Accordingly, a first aim of the current study was to explore the outcomes of the emotion regulation process by investigating the positive relationship between emotional dissonance and hostile customer relations that, in turn, are expected to report a positive association with the occurrence of mental health symptoms. Furthermore, the second aim of this research was to delve deeper into the buffering role of individual characteristics on the process of emotion regulation: in particular, proactive personality was considered as a protective factor that can help individuals to cope with managing emotions at work.

### 1.1. The Association between Emotional Dissonance and Mental Health

Over the past three decades, scholars have extensively investigated the dynamics involved in the process of emotion regulation [11]. Gross [12] described emotion regulation as the combination of a wide range of strategies that could be defined as corrective actions applied in order to modify unpleasant emotions (i.e., feelings) or their manifestations (i.e., expressions). The service sector typically requires the exhibition of positive emotional states, thus compelling workers to display positive emotions when feeling emotionally unresponsive, and to suppress negative affective states stemming from undesirable stimuli and events. A noteworthy literature review led Grandey and Melloy [13] to develop a comprehensive conceptual framework on how workers tackle and manage (i.e., regulate) their emotions. This model posits that the emotion regulation process is shaped by the dynamic interaction among variables belonging to the work environment, individuals and events. In order to manage and regulate their emotions, individuals can mainly adopt two strategy typologies that function during different moments of exposure to a stimulus. According to this model, the antecedent-focused strategies, also known as deep acting strategies, are based on the effort of altering one’s emotional state in order to truly experience the required emotion and, consequently, being genuine when performing the job. In contrast, the response-focused strategies, also referred to as surface acting strategies, entail the adjustment of the visible manifestation and expression of emotions, but do not modify the inner emotional experience [14]. The effort of regulating one’s perception of emotional events (i.e., antecedent-focused strategies) or the appearance of experienced emotional state (i.e., response-focused strategies) could have a significant impact on intrapsychic states, mainly in terms of emotional dissonance and energy depletion, as well as interpersonal performance, for instance through counterproductive work behaviours, organised societal behaviours, and emotional performance. These outcomes are momentary but may develop into long-lasting consequences concerning employees’ well-being (e.g., health symptoms, substance use, impaired sleep quality) and team/store performance (e.g., turnover, profits, customer loyalty). In other words, the immediate consequences of adopting emotion regulation strategies consist in intrapsychic and interpersonal results (proximal outcomes), which, over time, have an impact on secondary outcomes, essentially referring to well-being and performance (distal outcomes). Intrapsychic states included within the range of short-term outcomes of emotion regulation involve different levels of emotional dissonance, defined as the perceived discrepancy between required and experienced emotions [15]. To be specific, workers who are required to display emotions that are not genuinely felt must invest a great deal of effort that may gradually jeopardise their resources, primarily their emotional ones. This process could be framed within the Conservation of Resources Theory (COR theory) [16]. In short, COR theory postulates that individuals strive to obtain, retain, foster, and protect those things they centrally value (i.e., resources). According to this perspective, the costs of emotional dissonance entail an energy depletion process that reduces the amount of resources accessible in customer-facing activities. In line with the definition of loss spirals [17], it could be postulated that emotional dissonance could increase workers’ vulnerability to a further shortage of resources and, accordingly, lessen their ability to deal with customers efficiently. In this regard, perceived emotional dissonance was assumed to be associated with persistent adverse relationships with customers and these negative interactions, in the long run, are expected to jeopardise workers’ well-being and translate into greater mental health symptoms. This would be consistent with previous findings that indicate emotional dissonance as a main antecedent of harmful well-being and health conditions, such as emotional exhaustion and symptoms of depression [18,19]. Based on the theoretical framework and the empirical evidence described, the current study was aimed at exploring how emotional dissonance may be related to higher levels of mental health symptoms through the mediating role of impaired relationships with customers. Thus, the following hypothesis was formulated:

**Hypothesis 1.**  Hostile customer relations mediate the positive association between emotional dissonance and metal health symptoms.

### 1.2. The Buffering Role of Proactive Personality

To date, research on how workers’ characteristics could affect emotion management was mainly focused on sociodemographic characteristics (e.g., gender and social status), or personality traits [20,21]. In contrast, scholars call for a deeper understanding of the role that personal characteristics play in crafting the process of emotion regulation [13]. For instance, the scientific literature provides compelling evidence of the central role played by emotional intelligence in emotion regulation processes. In particular, emotional intelligence entails the individual ability to recognize appropriately different emotions and to use emotional information to manage one’s behaviour and thoughts [22]. Additionally, empirical evidence suggests that a greater emotional intelligence leads to adaptive regulation strategies that allow effective and positive management of one’s own emotions [23,24].

Grandey and Melloy [13] suggest focusing on individual differences that affect how workers fit the higher-order context, and especially within the working environment. In this regard, proactive personality has been defined as a relatively constant behavioural tendency to initiate change in the surrounding environment [25]. Thus, it constitutes an individual characteristic that allows workers to actively interact with the environment and shape it in order to better fit in with one’s work [26]. Yet there is compelling evidence from psychology research to show that proactive personality is associated with different positive outcomes for the individuals at their workplace, such as work engagement, organisational identification, job performance, and career success [27,28]. These findings suggest that workers characterised by a strong proactive personality tend to constantly pursue and identify opportunities within their work context and take advantage of them, showing autonomy, initiative, and perseverance until they attain meaningful results. Most importantly, research has shown that a proactive personality is crucial in the service sector. More specifically, a proactive personality leads workers to pay attention to customers’ requests in order to detect their expectations and, consequently, enhance their ability to serve customers [29]. As suggested by Huo and colleagues [30], the adoption of a customer-oriented perspective translates into a greater confidence in one’s work role. Accordingly, willingness to impact on the surrounding environment can also positively affect how proactive workers communicate with customers, given their enhanced aptitude for sharing their thoughts in a clear and efficient manner. Consistent with the discussed theoretical and empirical literature, the current study assumed that the interaction between a proactive personality, in other words an individual characteristic, and emotional dissonance, defined as an intrapsychic outcome of emotion regulation, is related to the quality of relationships that service workers develop with customers. Accordingly, the following study hypothesis was tested:

**Hypothesis 2.**  Within the relationship between emotional dissonance, hostile customer relations, and mental health symptoms, a proactive personality moderates the relationship between emotional dissonance and hostile customer relations. In particular, the occurrence of hostile customer relations is expected to be lower when employees experiencing high emotional dissonance are characterised by high levels of proactive personality.

**Figure 1:**
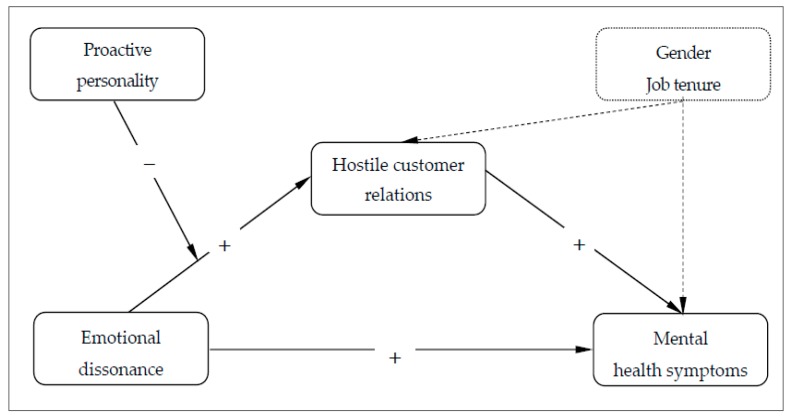
The hypothesised moderated mediation model.

The hypothesised moderated mediational model is depicted in Figure 1.

## 2. Methods

### 2.1. Procedure and Participants

Data were collected as part of a psychosocial risk assessment project carried out within one of the major Italian companies belonging to the large-scale retail sector. In agreement with the company’s Human Resources department, participants were asked to fill out a structured questionnaire during assisted compilation sessions where they could ask questions to one or two members of our research team. In particular, employees participated in short presentation sessions held by two members of the University research group aimed at presenting the general aim of the project, the procedures to collect the data and the complete absence of any potential risk or cost involved. Furthermore, the researchers informed the participants that the employer would not be informed of any decision not to complete the survey. The questionnaire included a statement regarding the personal data treatment, in accordance with the Italian privacy law (Law Decree DL-196/2003). Concerning the ethical standards for research, the study adhered to the latest version of the Declaration of Helsinki [31]. An additional ethical approval was not required since there was no treatment, including medical, invasive diagnostics or procedures causing psychological or social discomfort for the participants. Researchers invited all employees to fill out the paper-and-pencil questionnaire. The final sample consisted of *n* = 918 mass-retail employees. Most of them (62.7%) were women, and the mean age was 40.38 years old (SD (Standard Deviation ) = 7.68; min = 21; max = 60). Among them, 60.3% held a high school degree, 27.5% held a primary/secondary education degree, whereas the remaining 12.2% held a university degree. Most of the sample had a permanent employment contract (90.9%) and worked on a full-time basis (64.3%), and the mean organisational tenure was 13.21 years (*SD* = 8.87; min = 1; max = 40).

### 2.2. Measures

Emotional dissonance. The perceived discrepancy between emotions felt and those required by their professional role was measured using four items developed by Zapf and colleagues [32]. The Italian version of the scale was developed using a standard translation and back-translation procedure [33]. Sample items were: “How often does it occur in your job that you have to display emotions which do not correspond to inner feelings?” and “How often does it occur in your job that you have to display positive emotions while feeling indifferent?”. All items were scored on a four-point frequency scale, ranging from 1 = (almost) never to 4 = (almost) always. Higher scores imply that participants experience more frequently a discrepancy between authentic and displayed emotions. The internal consistency of the scale yielded a Cronbach’s alpha coefficient of α = 0.78.

Hostile customer relations. The frequency of conflicts and unfriendly interactions with customers was measured with a three-item scale adapted from the questionnaire developed by Guglielmi et al. [34]. Sample items were: “Interacting with customers is frustrating” and “I end up in arguments with customers”. Participants were asked to rate how often they experienced these situations on a five-point Likert scale ranging from 1 = never to 5 = very often. Accordingly, higher scores revealed a greater frequency of negative interactions with clients. Cronbach’s alpha for the scale was α = 0.65.

Proactive personality. The workers’ perception of their disposition to actively take ownership in their jobs and take personal initiative in order to modify their environment was assessed using six items taken from the Proactive Personality Scale [35,36]. Example items were: “I am always looking for better ways to do things” and “No matter what the odds, if I believe in something, I will make it happen”. All items were rated on a five-point Likert scale ranging from 1 = totally disagree to 5 = totally agree, with higher scores suggesting a higher perception of one’s proactive personality. In the present study, the reliability of the scale was α = 0.71.

Mental health symptoms. Poor mental health status was assessed using the 12-item version of the General Health Questionnaire (GHQ-12) [37,38]. Each item was rated on a four-point frequency scale, with higher scores indicating greater mental health symptoms. The current study employed the revised scoring procedure proposed by Goodchild and Duncan-Jones (C-GHQ) [39], as it demonstrated superior construct validity and greater sensitivity with respect to the traditional GHQ scoring method [40]. Based on this method, the scoring of negative items, such as “feeling unhappy and depressed” was 0, 1, 1, 1. The scoring of positive items, such as “being able to concentrate on whatever you’re doing” was 0, 0, 1, 1. Accordingly, the final score in mental health symptoms ranged between 0 and 12. The internal consistency of this scale was α = 0.80.

### 2.3. Strategy of Analysis

This exploratory study was aimed at performing a first attempt to explore the buffering role of proactive personality within the harmful relationship between emotional dissonance, hostile customer relations, and mental health symptoms. The study hypotheses were tested using the PROCESS macro developed by Hayes for SPSS [41]. To be specific, Model 4 was performed in order to test the mediating role of hostile customer relations in the association between emotional dissonance and mental health symptoms (Hypothesis 1). Model 7 was then used to verify the moderated mediational model (Hypothesis 2). Notably, in this model the path from the independent variable (i.e., emotional dissonance) to the mediator (i.e., hostile customer relations) was moderated by a fourth variable (i.e., proactive personality). The bootstrapping method provides robust estimates of standard errors and generates an estimate of the hypothesised effects, including a 95% confidence interval. According to this statistical resampling method, when zero does not fall into the confidence interval, the null hypothesis can be rejected. Both Hypothesis 1 and Hypothesis 2 were tested on 10,000 bootstrap re-samples. Moreover, gender and job tenure were included as control variables in the moderated mediational model.

## 3. Results

### 3.1. Descriptive Results

The means, standard deviations, internal consistencies, and correlations were computed for all study variables, as reported in Table 1. All significant relationships between the variables were in the expected direction. Internal consistency (Cronbach’s alpha) for all variables ranged between α = 0.65 and α = 0.80; thus, all scales reported an internal consistency value that met the criterion of 65 [42]. Emotional dissonance was positively related to hostile customer relations (r = 0.29; *p* < 0.001) and to mental health symptoms (r = 0.16; *p* < 0.001). Hostile relations with customers related negatively with proactive personality (r = −0.09; *p* < 0.01), but positively with the occurrence of mental health symptoms (r = 0.26; *p* < 0.001). Furthermore, proactive personality reported a negative association with the criterion variable of mental health symptoms (r = −0.19; *p* < 0.001).

### 3.2. Model Testing

The results from a simple mediation analysis indicated that, after controlling for gender and job tenure, emotional dissonance was indirectly related to mental health symptoms through its relationship with hostile customer relations. As reported in Table 2, emotional dissonance was positively related to hostile customer relations (*b* (SE, Standard Error) = 0.33 (0.04), *p* < 0.001, 95% CI, Confidence Interval (0.26; 0.40)), and hostile customer relations were subsequently and positively related to mental health symptoms (*b* (SE) = 0.65 (0.09), *p* < 0.001, 95% CI (0.47; 0.82)). A 95% confidence interval based on 10,000 bootstrap samples indicated that the indirect effect was entirely above zero (*b* (SE) = 0.21 (0.04), 95% CI (0.14; 0.29)). Approximately 10% of the variance in mental health symptoms was accounted for by the predictors (R^2^ = 0.10). These results supported the hypothesised mediating role played by hostile customer relations in explaining the relationship between emotional dissonance and mental health symptoms. Therefore, Hypothesis 1 was fully supported.

The upper part of Table 2 indicates that, after controlling for gender and job tenure, emotional dissonance was significantly associated with greater hostile customer relations (*b* (SE) = 0.33 (0.04), *p* = 0.000). On the contrary, proactive personality reported a negative association with hostile customer relations (*b* (SE) = −0.13 (0.04), *p* < 0.01). The dependent variable model (on the right of the upper part of Table 2) showed that hostile customer relations explained an additional and significant portion of mental health symptoms, after controlling for covariates and emotional dissonance, which turned into a non-significant path. Specifically, hostile customer relations were related to a higher prevalence of mental health symptoms (*b* (SE) = 0.65 (0.09), *p* < 0.001). This means that emotional dissonance was related to more hostile customer relations which, in turn, reported a positive association with general health symptoms. Finally, as shown in Table 2, we also tested the moderation between emotional dissonance and proactive personality on hostile customer relations, and we found that there was an additional, significant and negative effect of the interaction term (*b* (SE) = −0.12 (0.05), *p* = 0.02). Moreover, the index of moderated mediation was also significant (*b* = −0.08 Boot SE (0.03), 95% CI (−0.15; −0.02)). The lower part of Table 2 reports critical values of the conditional indirect effect.

The indirect relationship between emotional dissonance and mental health symptoms through hostile customer relations was tested when proactive personality (the moderator) was low (one standard deviation below the sample mean), medium (the sample mean), and high (one standard deviation above the sample mean). The obtained results suggest that the indirect association between emotional dissonance and mental health symptoms through hostile customer relations was significant at low (−1 SD; *b* (SE) = 0.42 (0.05), 95% CI (0.32; 0.52)), medium (Mean; *b* (SE) = 0.33 (0.04), 95% CI (0.26; 0.40)), and high levels of proactive personality (+ 1 SD; *b* (SE) = 0.24 (0.05), 95% CI (0.14; 0.34)).

**Figure 2:**
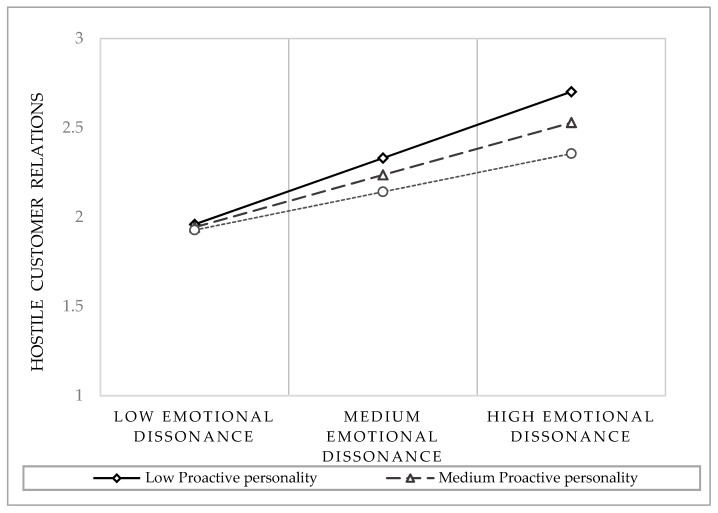
Interaction effect between emotional dissonance and proactive personality on hostile customer relations.

Figure 2 plots the nature of this interaction, showing that emotional dissonance has a higher effect on hostile customer relations, particularly among respondents who reported low levels of proactive personality, while it had a lower effect among those who reported relatively high levels of proactive personality. Therefore, Hypothesis 2 was also supported. Considering the control variables investigated, we found only gender to have an effect; to be specific, women reported higher levels of mental health problems (*b* = −0.76, *p* < 0.001), whereas there were no gender differences in terms of the relationship with customers. Additionally, no significant effects were found for job tenure.

## 4. Discussion

The aim of the current research was to gain a deeper insight in the process that links emotional dissonance and workers’ health in the service sector, through a closer examination of the role played by hostile customer relations as a mediating variable. Furthermore, a proactive personality was considered a personal resource capable of weakening the positive association between emotional dissonance and negative relationships with customers. Through a moderated mediation model, the present study contributed to research on those factors that may influence the emotion regulation and prevent a negative impact on employees’ health. In line with the assumptions of loss spirals postulated by the COR theory [16], the effort required and experienced in a divergence between experienced and expressed emotions could reduce the resource pool available for handling positive relationships with customers.

This evidence agrees with previous findings concerning the opposite outcomes for emotion regulation strategies based on reappraisal (i.e., the re-evaluation of emotional-eliciting stimuli in unemotional terms) and suppression strategies (i.e., based on the inhibition of emotional expressive behaviours). In contrast with reappraisal strategies, the suppression of one’s emotions results in lower levels of job satisfaction, well-being, and greater emotional exhaustion [43].

Consistently, the current results suggest that the mechanism of suppressing genuine emotions boost perceived frustration resulting from difficult customer interactions, which consequently leads to workers being mistreated by or arguing with customers.

The crucial role of contact with customers was highlighted by previous findings that recognized this characteristic of work in service organizations as the main antecedent of job burnout, as well as a moderator of the negative impact of emotional labour on exhaustion [44].

According to the present study, the dissonance between felt and displayed emotions enhances the likelihood to experience hostile relations that, in turn, are positively associated with mental ill-health conditions (i.e., mental health symptoms). This result corroborated the evidence that the experience of emotional dissonance among workers in client-driven environments could drain their resources and foster emotional exhaustion, which is subsequently associated with higher rates of certified sickness absence [45]. Moreover, the obtained results provided empirical support for the emotion regulation model developed by Grandey and Melloy [13]. Emotional dissonance emerged as an intrapsychic condition that is associated with an interpersonal outcome (i.e., hostile customer relations). As predicted by the model, in the current sample these short-term outcomes were negatively associated with workers’ well-being. Problematic interactions with customers affect workers’ health, as underlined by scholars who identify customer aggression as a strong predictor of an adverse health condition such as job burnout [46].

Additionally, our results concurred with previous evidence that frontline service staff are constantly required to regulate their emotions and comply with organisational rules and customers’ expectations and are therefore at higher risk of consuming their energies and becoming exhausted [47]. Zapf, Seifert, Schmutte, and Mertini [48] defined emotional dissonance as a specific type of role conflict that is associated with depersonalisation. In this sense, we could also interpret the mediating role of hostile customer relations as a consequence of a perceived emotional detachment derived from workers’ emotional labour. The obligation of displaying requisite emotions during social interaction could require a persistent emotional overextension which may lead to symptoms of depersonalisation, triggering an impersonal and unsuccessful response to customers’ needs [49]. Therefore, workers experiencing high levels of emotional dissonance also perceive customer relations as frustrating and problematic. On the other hand, the association between emotional dissonance and customer relations could be buffered by specific individual characteristics. In particular, the second aim of the current study was to support the moderating role of proactive personality between emotional dissonance and hostile customer relationships. The active role assumed by workers in their attempt to regulate their attitude towards work activities allows them to have greater control in structuring and changing their situations, and to persevere until such change occurs [50]. In this sense, employees who are less proactive are more likely to adapt to situations, suffer the consequence of emotional dissonance, and give up actively dealing with the customer. On the contrary, rather than adapting to present situations, workers who are more proactive are able to adjust their performance in order to fit the job role, confronting emotional labour differently, and are likely more effective in interacting with customers [51]. Consequently, it has been perceived that relationships with customers were more constructive and had positive consequences for workers’ well-being. In line with earlier research, a proactive personality requires a propensity for the application of successful strategies with the goal of managing responses to discrepancies [52]. In other words, workers with a proactive personality actively adapt their emotional strategies in accordance with organisational goals or, within the service sector, to what is required in order to achieve a functional customer relationship. Overall, proactive employees are better able to control their negative inner emotions (in addition to their visible expression) when interacting with customers and to understand customers’ needs, with the result of establishing positive relationships with customers, consequently safeguarding their company’s image as well as their own well-being.

### 4.1. Strengths, Limitations, and Future Research Directions

To our knowledge, the current study was one of the earlier attempts at investigating the role of a proactive personality as a buffer against harmful consequences of emotional dissonance, and was thus a significant contribution to the field of emotion management at work. Furthermore, the number of participants was considerable and provided support to the statistical validity of our findings. Nevertheless, the study had some limitations that should be mentioned. On the one hand, study variables were assessed using a self-report questionnaire and were rated by the same data source. As such, common method variance may affect the relations between constructs [53]. On the other hand, while hostile customer relations and mental health symptoms could also be assessed also using objective measures, emotional dissonance and proactivity are intrinsically subjective. Hence, the employment of self-report measures was the most rational approach to exploring these variables. As a further limitation, the cross-sectional nature of this study prevented us from determining the direction of the causal relationship between emotional dissonance and hostile customer relations. It may be argued that managing difficult relationships with clients could lead to the onset of emotional states that diverge from those imposed by the workplace requirements, thus resulting in higher levels of emotional dissonance, as suggested by earlier research findings [54]. As such, the use of a multi-wave study design would establish the actual direction of this association and, hopefully, explore the occurrence of reciprocal relationships between emotional dissonance and hostile customer relations. Additionally, the sample consisted of employees working in the mass-retail distribution sector, therefore focusing on a specific portion of customer-facing professions. Future research should assess if this empirical evidence could be extended to additional work roles involving customer relations (e.g., tourism sector).

Furthermore, this study focused on emotional dissonance as the outcome stemming from the employment of an emotional regulation strategy that fakes the required emotional expressions (i.e., surface acting). Future research should also consider strategies intended to align one’s true emotions with those required by the work role (i.e., deep acting) in order to provide an exhaustive insight on the compound association between opposite emotional labour strategies and the relations between frontline workers and customers.

### 4.2. Practical Implications

Previous research has highlighted that individual differences, such as proactive personality, are not the unique predictors of proactive behaviour, but rather they could be encouraged by contextual factors capable of promoting a proactive attitude [28,55]. In this sense, if on the one hand literature suggests that a selection approach helps to identify personal competences which influence workers’ active regulation of organisational relationships, on the other hand interventions related to organisational aspects could play a pivotal role. Concerning this second aspect, most of the research focused on job design (e.g., enrichment work, autonomy, goal setting), but also training and development approaches, which are sometimes overshadowed, can promote a proactive work force [56]. Training is important for making employees aware of the importance of personal expectations that allow them to improve career prospects, develop new skills, innovate content and working procedures, and seek out new information and feedback [57]. Particularly, if we considered proactivity as the possibility of reducing discrepancies between present contingency and values taken as a reference model, training interventions could help employees to increase awareness of their own reference values in order to identify possible behavioural actions and opportunities to achieve them [51]. Proactivity generally also implies a long-term vision which allows anticipating future outcomes, preparing for dealing with future problems, and exploring new opportunities [58]. Starting from this premise, other authors have developed proactivity training focused on skills related to the development of initiative, like innovativeness, proactive goals, planning, and time management [59]. However, the possibility of taking the initiative to improve current situations and create new opportunities depends on organisations. They should be proactive and delegate work activities to employees themselves. This organisational culture strives to reduce perceived barriers to change and perceived risk, and maximise perceived safety, allowing workers to engage in work-related proactivity [60]. From the perspective of human resources management, regular feedback from a superior, sharing objectives with workers, and an appreciation for initiative and leaders’ support [61] enables individuals to implement proactive conducts. Other research confirmed that organisational and social processes shape proactivity in terms of discussing ideas with managers, autonomy, and peer support, which enhance workers’ self-efficacy for proactivity and allows them to feel more confident about acting appropriately [62,63].

Based on the assumption that traits are elicited (or inhibited) as responses to situation cues, previous findings identified job autonomy as a crucial boundary condition to prompt the manifestation of proactive personality that, in turn, may enhance workers’ motivation and involvement towards their work (i.e., work engagement) [64].

The combination of training initiatives that foster individual skills linked to proactive attitudes, and HR management strategies that favour workers’ personal initiative, could shape an organisational environment that enables and encourages proactive service workers. Emotional dissonance can be significantly influenced by organizational policies. In particular, emotional display rules have a fundamental role in determining the perceived distance between felt emotions and those which have to be displayed [13]. Except for jobs characterized by high levels of emotional labour, where display rules are clearly shared with employees to improve their emotional performance, this may not apply to every organizational context [65]. This study underlines the relevance of emotional dissonance in managing customer relationship and its detrimental effects on individual health and well-being.

From an organizational viewpoint, sharing information with employees about the expected emotions displayed at work through organizational policies can help them to manage and to detect the effective discrepancies between felt and exhibited feelings. Furthermore, organizational policies should promote deep acting strategies which can help workers to better cope with emotion demands while working and, at the same time, to promote genuine relationships with customers.

## 5. Conclusions

This study suggests that, among customer-facing work roles, the perception of a constant divergence between emotions experienced and those required by the organisational context could jeopardise workers’ ability to interact with customers efficiently. On the other hand, proactive personality has emerged as an individual characteristic that enables workers to adopt suitable emotion management strategies, even when faced with great levels of emotional dissonance. This evidence could be strategic for developing training interventions devoted to individuals working in a sector where appropriate interactions with customers are pivotal for providing quality service.

## Figures and Tables

**Table 1:** Means, standard deviations, Cronbach’s alpha (on the diagonal) and correlations among study variables (*n* = 918).

Variable	Range	M	SD	*R*					
				1	2	3	4	5	6
1. Gender (1 = male)	0–1	0.37	0.48	*n.a.*					
2. Job tenure	1–40	13.21	8.87	0.13 ***	*n.a.*				
3. Emotional dissonance	1–4	2.47	0.89	−0.20 ***	−0.07 *	(*0.78*)			
4. Hostile customer relations	1–5	2.23	0.10	−0.06	−0.02	0.29 ***	(*0.65*)		
5. Proactive personality	1–5	3.66	0.73	0.07 *	−0.05	−0.01	−0.09 **	(*0.71*)	
6. Mental health symptoms	0–12	3.17	2.70	−0.15 ***	0.02	0.16 ***	0.26 ***	−0.19 ***	(*0.80*)

Note: * *p* < 0.05; ** *p* < 0.01; *** *p* < 0.001. M = Mean; *SD* = Standard Deviation.

**Table 2:** Results of the moderate-mediation model.

Variable	Path Coefficients							
	To Hostile Customer Relations			To Mental Health Symptoms				
	Coefficient	SE	Boot 95% CI	Coefficient	SE		Boot 95% CI	
Gender (1 = male)	0.01	0.07	−0.12; 0.14	−0.76 ***	0.18		−1.11; −0.40	
Job tenure	0.00	0.00	−0.01; 0.01	0.01	0.01		−0.01; 0.03	
Emotional dissonance	0.33 ***	0.04	0.26; 0.40	0.17	0.10		−0.03; 0.37	
Proactive personality	−0.13 **	0.04	−0.22; −0.04					
Emotional dissonance Proactive personality	−0.12 *	0.05	−0.21; −0.03					
Hostile customer relations				0.65 ***	0.09		0.47; 0.82	
Model Summary	R^2^ = 0.10 ***			*R*^2^ = 0.10 ***				
Conditional indirect relationship between emotional dissonance (X) and mental health symptoms (Y) through hostile customer relations (M) at values of proactive personality (W).								
	Effect	Boot SE			Boot 95% CI			
Low Proactive personality	0.27	0.05				0.17; 0.38		
Medium Proactive personality	0.21	0.04				0.14; 0.29		
High Proactive personality	0.16	0.04				0.09; 0.23		

Note: * *p* < 0.05; ** *p* < 0.01; *** *p* < 0.001. SE = Standard Error; Boot 95% CI = Bootstrapped 95% Confidence Interval.
